# Hgtsynergy: a transfer learning method for predicting anticancer synergistic drug combinations based on a drug-drug interaction heterogeneous graph

**DOI:** 10.1186/s12859-025-06360-5

**Published:** 2026-01-06

**Authors:** Xiaowen Wang, Yanming Huang, Hongming Zhu, Dongsheng Mao, Xiaoli Zhu, Qin Liu

**Affiliations:** 1 School of Computer Science and Technology, Tongji University, Shanghai, 201804 China; 2 Department of Clinical Laboratory Medicine, Shanghai Tenth People’s Hospital, School of Medicine, Tongji University, Shanghai, 200072 China

**Keywords:** Drug synergy prediction, Heterogeneous graph attention network, Drug-drug interaction, Transfer learning, Deep learning

## Abstract

****Background**:**

Drug combination therapy often outperforms monotherapy in cancer treatment, but the vast number of available drugs makes manual screening for synergistic combinations costly. Computational methods, especially deep learning, can reduce the search space by predicting likely synergistic drug combinations. Recent studies have improved drug synergy prediction by modeling associations among different biological entities, but drug–drug interactions have not been fully leveraged in this scenario, which motivated the work presented in this paper.

****Methods**:**

This paper proposes a deep learning method named HGTSynergy to predict synergistic drug combinations, which employs a heterogeneous graph attention network and a tailored task to capture complex latent patterns in the drug network as prior knowledge. The learned knowledge is then transferred through a transfer learning framework to the downstream task of predicting drug synergy scores, effectively enhancing predictive performance.

****Results**:**

A five-fold nested cross-validation is employed to train HGTSynergy. In the synergy regression task, HGTSynergy outperforms seven deep learning methods, achieving a mean squared error of 222.83, root mean squared error of 14.91, and Pearson correlation coefficient of 0.75. For the synergy classification task, it also surpasses other methods with an area under the receiver operating characteristic curve of 0.90, area under the precision–recall curve of 0.63, accuracy of 0.94, precision of 0.72, and Cohen’s Kappa of 0.52. The ablation study verifies that the heterogeneous graph attention network and the transfer learning framework both have a positive effect on prediction performance. Moreover, a series of analyses demonstrates that the proposed method exhibits strong generalization performance and interpretability. The case study further validates its consistency with prior research.

****Conclusions**:**

This study suggests that drug synergy prediction can be improved by comprehensively modeling diverse drug–drug interaction types and leveraging transfer learning to extract prior knowledge from them. The ability of HGTSynergy to discover new anticancer synergistic drug combinations outperforms other state-of-the-art methods. HGTSynergy promises to be a powerful tool to pre-screen anticancer synergistic drug combinations.

**Supplementary Information:**

The online version contains supplementary material available at 10.1186/s12859-025-06360-5.

## Background

Cancer drug therapy is a major challenge facing humanity today. Drug combination therapy is regarded as the foundation of cancer treatment [1] since it usually has advantages over monotherapy, such as better efficacy [2, 3], lower side effects [4], and lower possibility of drug resistance [5]. The effects of drug combinations on cell lines include synergy, antagonism, and additivity [6, 7], meaning that the effect of a drug combination can be greater than, less than, or equal to the sum of the effects of each drug used individually. Therefore, drug combinations are not always more beneficial, which makes the task of identifying synergistic drug combinations particularly meaningful.

In the early days, synergistic drug combinations were mainly identified based on clinical experience. This approach has significant drawbacks, not only requiring a large amount of manpower and time but also potentially exposing patients to unnecessary or even harmful treatments [8]. In modern medical scenarios, the rapidly growing number of anticancer drugs has exponentially expanded the search space for potential drug combinations, further amplifying the limitations of relying solely on clinical experience to screen drug combinations.

In recent years, researchers in the biological field have employed automated methods to conduct experiments, using high-throughput screening (HTS) techniques to perform many experiments and accumulate anticancer drug synergy data. Currently, several publicly available drug synergy datasets are available for researchers, which record the response effects of different drug combinations on various cell lines, laying the foundation for using machine learning (ML) methods to predict drug synergy.

With the development of ML, models such as linear regression, support vector machines, and random forests have been proposed, and many studies [9–11] have begun to use these ML models to predict synergistic drug combinations. For example, H-RACS proposed by Yang et al. [11] constructed features for drug combinations and cell lines using data including genes, drug chemical structures, protein interaction networks, and cancer pathways, and used a gradient boosting machine to predict synergy scores. However, traditional ML methods are overly dependent on feature engineering, limited by expert experience, and are not effective enough at uncovering latent patterns in the data.

Recently, with the rise of deep learning (DL), feedforward neural networks (FNNs) have emerged in drug synergy prediction. DeepSynergy [12], MatchMaker [13], MARSY [14] employed FNNs to learn latent features of drugs and cell lines, and then predicted drug synergy scores based on these features, achieving better performance than conventional ML methods. Although the application of FNNs has improved performance, FNNs still lack generalization ability and require large amounts of training data due to numerous parameters.

Earlier drug synergy prediction models based on FNNs primarily focused on extracting intrinsic features of the biological entities. In contrast, recent studies have begun to incorporate relational information between biological entities by introducing models with additional structural components. One important model is the graph neural network (GNN). Compared to FNNs, GNNs have fewer parameters and better generalization performance in some cases. Moreover, in bioinformatics, GNNs are suitable for training on non-Euclidean data, which is common when representing relationships between biological entities. Based on GNNs, researchers can enhance the performance of drug synergy prediction by incorporating hidden information of biological relationships. DeepDDS [15], HypergraphSynergy [16], PRODeepSyn [17], HANSynergy [18], PathSynergy [19] employed GNNs to capture associations among various biological entities and thus enhance drug synergy prediction performance. Additionally, the Transformer architecture is also well-suited to capturing relationships among biological entities due to its self-attention mechanism, which enables the model to weigh interactions among all entities simultaneously and to model long-range dependencies in the data. TranSynergy [20], DTSyn [21], GraphTranSynergy [22] employed Transformer architectures to capture relationships among biological entities and achieved prediction of synergistic drug combinations.

Although many studies have incorporated additional interaction datasets and designed models to capture associations among various biological entities to enhance synergy prediction, studies that introduce additional drug-drug interaction (DDI) datasets remain limited. However, DDIs represent critical associations that should be carefully considered in combination therapies, which is because during drug combination therapy, DDIs do not always align with expectations owing to the complexity of their underlying biochemical mechanisms [23], which hinders further improvement of drug synergy prediction. Therefore, thoroughly considering the impact of DDIs in the synergy prediction task can help the model better understand the complex biochemical interactions between drugs, thereby further improving drug synergy prediction. Currently, some studies have introduced additional DDI datasets and taken the influence of DDIs into account in drug synergy prediction. Wang et al. [24] incorporated DDI data from the DrugBank database and treated DDI binary classification as a subtask within a multi-task learning framework, using an FNN to classify each DDI as positive or negative, thereby improving drug synergy prediction performance. However, the mechanisms of drug interactions are highly complex, so dividing DDIs into only two categories may oversimplify the problem. Monem et al. [25] also incorporated DDI data from the DrugBank database and divided DDIs into plenty of categories, employing an FNN to construct a pre-training network for predicting DDI categories to learn prior knowledge of DDI information, thereby fusing the DDI information with cell line features to enhance drug synergy prediction performance. Although further considering plenty of categories of DDIs, FNNs may fail to capture latent DDI patterns effectively due to the limited capacity for relation fitting. To conclude, simplified object descriptions (i.e., drug-association class descriptions) or model construction may hinder further improvements in drug synergy prediction due to insufficient cross-disciplinary information or knowledge.

To solve the above limitations, this paper proposes a drug synergy prediction framework named HGTSynergy (A **H**eterogeneous-**G**raph-Based **T**ransfer Learning Method for Drug **Synergy** Prediction). On one hand, HGTSynergy constructs the drug network as a heterogeneous graph and uses different meta-paths to represent different DDI types. The proposed method differs from previous studies based on heterogeneous graphs [18, 26], which primarily focused on the relationships between different types of biological entities when constructing the heterogeneous graph, while overlooking the diversity of relationships among entities of the same type. In contrast, the heterogeneous graph proposed in this paper explicitly models the multi-type DDIs between drug nodes, enabling more fine-grained representation of interactions among drugs. On the other hand, HGTSynergy employs a transfer learning framework, which enables the model to learn prior knowledge from the large-scale DDI heterogeneous graph through a tailored pre-training task. Based on this, abundant latent information of DDIs can be transferred into the downstream synergy prediction network and play a positive role. The experiments are conducted on the O’Neil dataset, and the results demonstrate that HGTSynergy outperforms other state-of-the-art (SOTA) methods. Ablation studies and a series of analyses are conducted to further verify the mechanisms of HGTSynergy in detail. Case studies indicate that the prediction results of HGTSynergy are consistent with many previous research findings. In summary, HGTSynergy demonstrates strong performance in drug synergy prediction and holds promise as a powerful tool for predicting synergistic combinations of anticancer drugs.

## Materials and methods

### Synergy dataset

O’Neil dataset [4] is a synergy dataset including 38 drugs and 39 cell lines from 7 tissues, with experimentally measured cell growth rates of each cell line under different drug combinations. More details of O’Neil dataset are presented in Additional file 4: S3. The experiments utilize Loewe Additivity [27] and Bliss Independence [28] synergy scores computed based on the O’Neil dataset as prediction labels. Loewe synergy scores are obtained from [12] with 23,052 samples, and we average scores for repeated drug pair and cell line combinations to obtain 22,737 samples. Bliss synergy scores are obtained from [29]. In the collected synergy score data, each sample is a quadruple in the form of (Drug A, Drug B, Cell Line, Synergy Score), describing the synergistic effect of a drug pair on a specific cell line. In line with common practice in [12], the samples are divided into five folds according to the Leave Drug Combinations Out strategy, which ensures that each drug combination appears only in one fold, facilitating the evaluation of the model’s generalization ability to novel drug combinations through cross-validation.

### Feature construction

Cell line features are constructed following the approach proposed by Wang et al. [17] by using gene expression levels obtained from the ArrayExpress database [30], gene mutations from the COSMIC database [31], and protein-protein interaction (PPI) relationships from the STRING database [32]. Then, it integrates the PPI network with omics data via a graph convolutional network (GCN) to generate low-dimensional dense embeddings for cell lines. The detailed information of cell lines used in this work is presented in Additional file 1, and detailed steps for constructing cell line features in [17] are presented in Additional file 4: S2.

To construct drug features, drug information is obtained from DrugBank (v5.1.12) [33], and drugs are filtered based on their categories, retaining only legal drugs intended for human use. Subsequently, SMILES representations for 11,719 drugs are obtained, as presented in Additional file 2. Then, the RDKit toolkit [34] is employed to convert SMILES representations into drug features, which are composed of Morgan fingerprints, MACCS fingerprints, and molecular descriptors. Specifically, Morgan fingerprints with a radius of 3 are generated and encoded as a 1024-dimensional binary vector for each drug. Additionally, MACCS fingerprints are generated, resulting in a 167-dimensional binary vector for each drug. Molecular descriptors with missing values are filtered out, yielding a 196-dimensional vector for each drug. The three types of vectors are then concatenated and subjected to z-score normalization, resulting in a 1387-dimensional feature vector for each drug. Additionally, due to the high dimensionality of the original drug features, an autoencoder is trained on the features of the 11,719 drugs and subsequently used to project them into a 128-dimensional latent space. Specifically, an autoencoder comprises an encoder and a decoder: the encoder maps the input to a low-dimensional latent representation, and the decoder reconstructs the original input from this compressed representation. The encoder and decoder are trained jointly in an end-to-end manner, and the trained encoder is subsequently used for dimensionality reduction of drug features. Both components consist of multiple fully connected layers with the ReLU activation function and share a symmetric architecture, which can be formulated as:

1 $$\begin{aligned} \begin{aligned} \text {AE-Encoder}(\textbf{x},n)&= \text {FC}_n \left( \text {ReLU} \left( \text {FC}_{n-1} \left( \cdots \text {ReLU}( \text {FC}_1(\textbf{x})) \cdots \right) \right) \right) \\ \text {AE-Decoder}(\textbf{x},n)&= \text {FC}'_n \left( \text {ReLU} \left( \text {FC}'_{n-1} \left( \cdots \text {ReLU}( \text {FC}'_1(\textbf{x})) \cdots \right) \right) \right) \end{aligned} \end{aligned}$$

where *n* denotes the number of network layers, and the *i*-th layer $\text {FC}_i$ and $\text {FC}'_i$ in the encoder and decoder are symmetric in architecture. Thus, the generation process for the feature of drug *d* can be denoted as $\textbf{x}_d^{\text {drug}} = \text {AE-Encoder}([\textbf{x}_d^{\text {morgan}} \parallel \textbf{x}^{\text {maccs}}_d \parallel \textbf{x}^{\text {descriptors}}_d], n_1)$ with $\parallel$ denoting the concatenation operation and $n_1$ denoting the number of layers of the autoencoder dimensionality reduction module.

### Drug-drug interactions

**Fig. 1:**
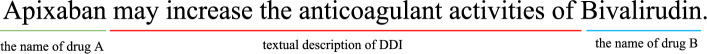
An example of breaking down a DDI sentence

A total of 1,094,569 sentences describing each drug–drug interaction (DDI) are obtained from DrugBank (v5.1.12), with all interactions restricted to legal drugs intended for human use, as previously described. Based on these DDI sentences, an approach similar to that of [35, 36] is adopted to transform the textual descriptions into categorical DDI data. Specifically, each DDI sentence can be broken down into three parts: the name of drug A, the name of drug B, and the textual description of DDI. Figure 1 is an example showing how to break down a sentence, where “Apixaban” is the name of drug A, “Bivalirudin” is the name of drug B, and the remaining part “may increase the anticoagulant activities of” is the textual description of DDI between the two drugs. After processing, DDI sentences can be denoted as (Drug A, Drug B, DDI textual description), equivalently expressed as (Drug B, Drug A, DDI textual description). Then, identical textual descriptions of DDIs can be considered as the same DDI category, while different textual descriptions of DDIs can be regarded as different DDI categories. According to this processing rule, DDIs are classified into 293 categories. All DDI descriptions used in this work are presented in Additional file 3.

### HGTSynergy

#### Transfer learning framework

HGTSynergy is built on a transfer learning framework, which is an ML approach that aims to improve model performance in a new task by utilizing knowledge learned from a related task. This approach is inspired by how humans learn, using prior knowledge and experience to help with new learning tasks. Specifically, HGTSynergy includes a tailored pre-training task to learn prior knowledge from the large-scale DDI dataset to help the model achieve better prediction performance on the small-scale synergy score dataset. In practical applications, the learning process of HGTSynergy can be divided into two stages: pre-training and fine-tuning.

*Pre-training stage*: Large-scale DDI classification samples from DrugBank are used to pre-train the model on the DDI classification task. The pre-training stage enables the model to capture the interaction information among numerous drugs and store this prior knowledge in its parameters, thereby establishing a solid foundation for the subsequent task.

*Fine-tuning stage*: During the fine-tuning stage, the parameters of the pre-trained DDI encoder are transferred to the downstream synergy prediction model, thereby leveraging the DDI knowledge learned from the pre-training task. This process optimizes the model performance on the synergy prediction task using the small-scale synergy score dataset.

Through the proposed transfer learning pipeline, the model is able to acquire knowledge from a new perspective. Specifically, the downstream synergy prediction task focuses on capturing the ternary associations among (Drug A, Drug B, Cell Line). In contrast, pre-training on the DDI dataset encourages the model to learn binary associations between (Drug A, Drug B) using an alternative type of auxiliary data. As a result, the pre-training stage captures complex relationships between drugs and stores this binary relational information in the network parameters, thereby providing a complementary perspective that enhances the performance of the downstream synergy prediction task.

**Fig. 2:**
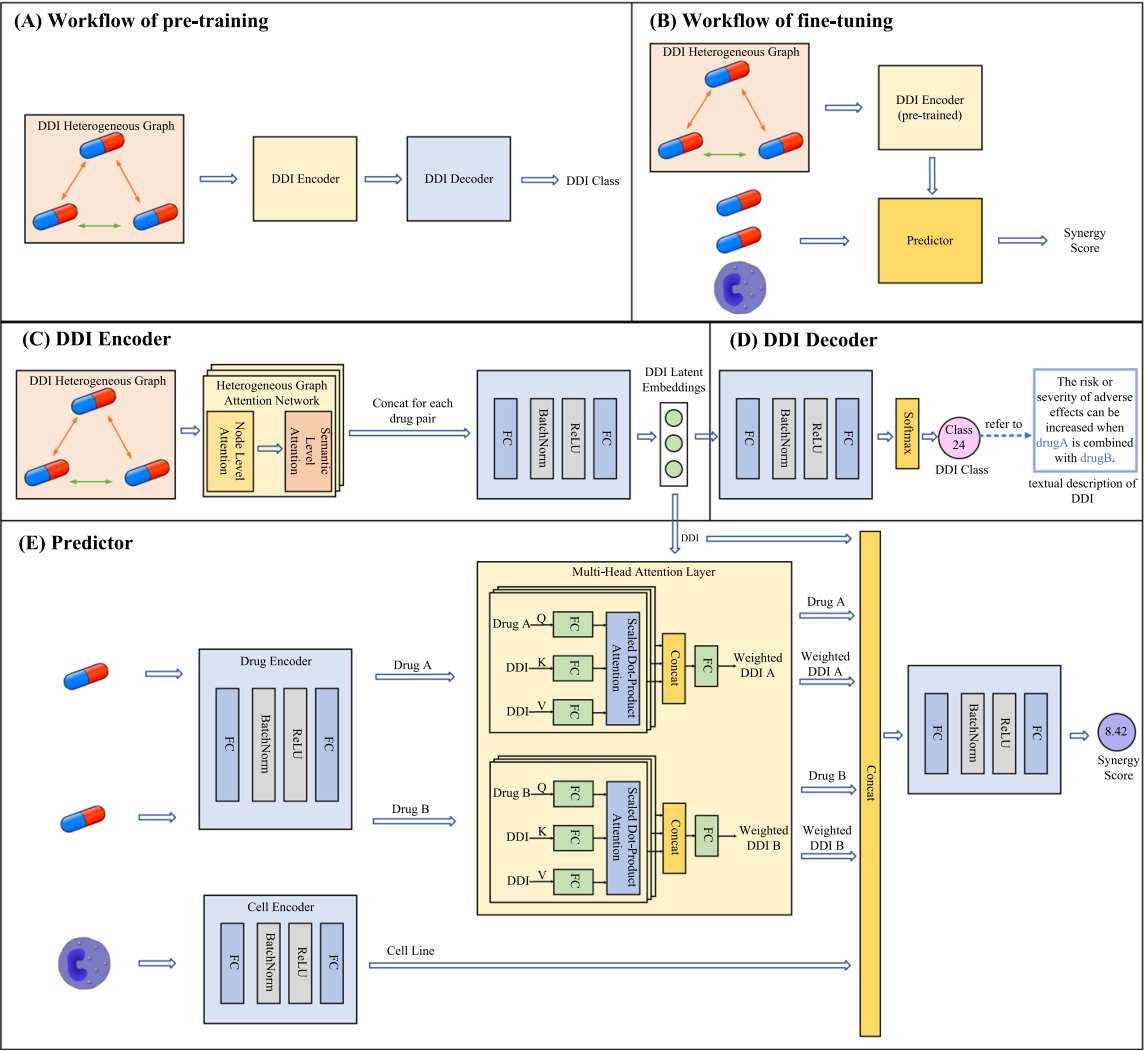
Overview of HGTSynergy

#### Model architecture

Based on the aforementioned transfer learning strategy, this paper proposes an overall architecture for drug synergy prediction, as illustrated in Fig.  2. The analysis of model complexity is presented in Additional file 4: S5. The model consists of two main phases: a pre-training phase (Fig.  2A) and a fine-tuning phase (Fig.  2B).

In the pre-training phase, a DDI classifier is designed based on an Encoder-Decoder architecture. Specifically, DDI Encoder compresses input information into a latent space, while DDI Decoder performs the classification task. The goal of this pipeline is to optimize the latent space during pre-training. To better capture drug–drug relationships in DDI classification, a large-scale heterogeneous DDI graph is constructed. DDI Encoder then employs a heterogeneous graph attention network (HAN) [37] to aggregate information from different drug nodes, followed by a multi-layer perceptron (MLP) that compresses and maps the aggregated features into a unified latent space, yielding the DDI latent embeddings. DDI Decoder subsequently decodes these embeddings via another MLP to predict DDI categories using the information in the latent space. This Encoder-Decoder design enables the model to optimize the latent space under the supervision of the DDI classification task and learn to generate high-quality DDI latent embeddings.

In the fine-tuning phase, the parameters of DDI Encoder are retained and further trained jointly with the downstream Predictor network. The model simultaneously leverages both cell line and drug features, as well as the DDI latent embeddings generated by the pre-trained model for predicting synergy scores. Specifically, Predictor first maps the cell line and drug features into the same latent space as the DDI embeddings using MLP layers. Then, a multi-head attention mechanism is used to integrate and fuse the drug features with the DDI embeddings. Finally, all features are concatenated and fed into the final MLP to predict drug synergy scores.

#### DDI encoder

DDI Encoder is shown in Fig.  2C, which is the core module of HGTSynergy in the transfer learning process. A DDI heterogeneous graph is constructed from numerous DDIs, and DDI Encoder based on HAN is employed to update node embeddings and generate latent representations for each drug pair for subsequent network use.

*DDI graph construction:* To model heterogeneous interactions in the drug network, a heterogeneous graph is introduced to construct drug relationships. The definition of a heterogeneous graph is given by:

##### Definition 1

(*Heterogeneous Graph*) [37] A heterogeneous graph, denoted as $\mathcal {G}=(\mathcal {V},\mathcal {E})$, consists of a node set $\mathcal {V}$ and an edge set $\mathcal {E}$. A heterogeneous graph is also associated with a node type mapping function $f_{n}: \mathcal {V}\xrightarrow {}\mathcal {A}$ and an edge type mapping function $f_{e}: \mathcal {E}\xrightarrow {} \mathcal {R}$. $\mathcal {A}$ and $\mathcal {R}$ denote the sets of predefined node types and edge types, where $|\mathcal {A}|+|\mathcal {R}|>2$.

**Fig. 3:**
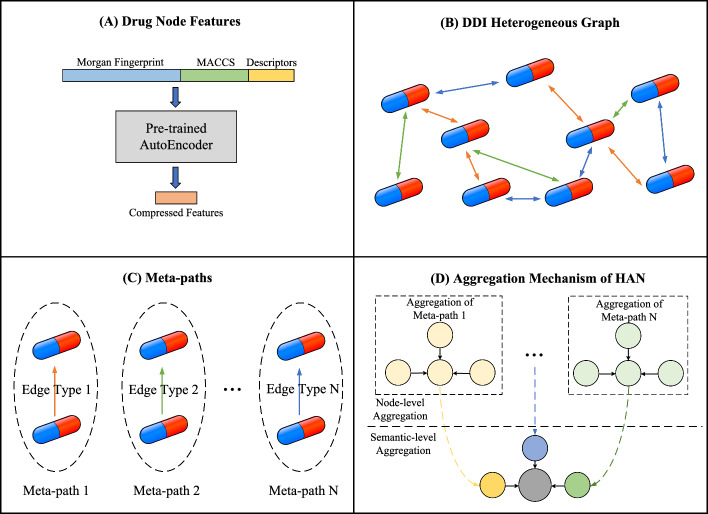
Construction of DDI heterogeneous graph and the mechanism of HAN

Then, the node type set and the node set can be formulated as:

2 $$\begin{aligned} \begin{aligned} \mathcal {A}&= \{a_1\} \\ \mathcal {V}&= \{d_1, \cdots , d_{n_{\text {drug}}}\} \\ \end{aligned} \end{aligned}$$

where $n_{\text {drug}}$ denotes the number of drugs. Specifically, the node set of the heterogeneous graph is identical to the drug set, where each node represents a drug and is associated with node features defined in section “Feature construction”, as illustrated in Fig.  3A. Besides, all nodes are defined as having the same type, i.e., $|\mathcal {A}| = 1$.

Additionally, the edge type set and the edge set can be formulated as:

3 $$\begin{aligned} \begin{aligned} \mathcal {R}&= \{r_1, r_2, \ldots , r_P\} \\ \mathcal {E}^{(i)}&= \{\{u, v\} \subseteq \mathcal {V} \mid \text {drugs } u \text { and } v \text { have a DDI of type } r_i\}, \quad i = 1, \dots , P \\ \mathcal {E}&= \bigcup _{i=1}^{P} \mathcal {E}^{(i)} \end{aligned} \end{aligned}$$

where *P* represents the number of DDI categories. Specifically, each edge represents a DDI related to two drug nodes, which is undirected since the order of drugs in a combination does not affect the DDI category. Besides, each edge type corresponds to a certain DDI category, thus $|\mathcal {R}| = P$. Based on these elements, the DDI heterogeneous graph can be constructed, as shown in Fig.  3B.

Additionally, in a heterogeneous graph, two objects can be connected through different semantic paths, referred to as meta-paths, defined as:

##### Definition 2

(Meta-path) [37] A meta-path $\phi _i$ is defined as a path in the form of $A_1 \xrightarrow {R_1} A_2 \xrightarrow {R_2} \cdots \xrightarrow {R_l} A_{l+1}$, which describes a composite relation $R=R_1 \circ R_2 \circ \cdots R_l$ between objects $A_1$ and $A_{l+1}$, where $\circ$ denotes the composition operator on relations.

Specifically, HGTSynergy models meta-paths by considering only one-hop connections, as illustrated in Fig.  3C. Each meta-path can be formulated as:

4 $$\begin{aligned} \phi _i = (a_1 \xrightarrow {r_i} a_1), \quad r_i \in \mathcal {R} \end{aligned}$$

where $\phi _i$ denotes a meta-path corresponding to a specific DDI relation type $r_i$. These meta-paths describe one-hop connections between drug nodes, and serve as the basis for relation-aware message aggregation.

*Heterogeneous graph learning:* Given the construction of the DDI heterogeneous graph, the HAN with *L* layers is applied to update node embeddings by integrating neighboring node embeddings through meta-paths. As shown in Fig.  3D, HAN first fuses the features of nodes along each meta-path separately through a node-level attention mechanism, and then integrates the features of nodes along different meta-paths through a semantic-level attention mechanism, thereby achieving heterogeneous information fusion. First, we define the initial embedding of each node *u* as the drug feature constructed in section “Feature construction”:

5 $$\begin{aligned} \textbf{h}_{u}^{0} = \textbf{x}_u^{\text {drug}} = \text {AE-Encoder}([\textbf{x}_u^{\text {morgan}} \parallel \textbf{x}_u^{\text {maccs}} \parallel \textbf{x}_u^{\text {descriptors}}], n_1) \end{aligned}$$

Subsequently, the initial node embeddings are updated through *L* layers of the HAN model. At each layer *l*, node-level aggregation is first performed, focusing only on meta-path based neighbors as defined:

##### Definition 3

(*Meta-path Based Neighbors*) [37] Given a node *u* and a meta-path $\phi _i$ in a heterogeneous graph, the meta-path based neighbors $\mathcal {N}_{u,\phi _i}$ of node *u* are defined as the set of nodes that connect with node *u* via meta-path $\phi _i$.

Specifically, in the DDI graph, for a given node *u* and a meta-path $\phi _i = (a_1 \xrightarrow {r_i} a_1)$, the meta-path based neighbor set is defined based on the typed edge set $\mathcal {E}^{(i)}$:

6 $$\begin{aligned} \mathcal {N}_{u,\phi _i} = \{ v \in \mathcal {V} \mid \{u, v\} \in \mathcal {E}^{(i)} \} \end{aligned}$$

To differentiate the degrees of relevance among different drug nodes, attention weights are assigned to neighboring nodes during node-level aggregation. For a given node *u* and its meta-path $\phi _i$ based neighbor *v*, the attention score is computed as:

7 $$\begin{aligned} e_{uv,\phi _i}^{l} = \sigma \left( [\textbf{a}_{\phi _i}^{l}]^{\top } \cdot \left[ \textbf{h}_{u}^{l-1} \parallel \textbf{h}_{v}^{l-1}\right] \right) \end{aligned}$$

8 $$\begin{aligned} \alpha _{uv,\phi _i}^{l} = \frac{\exp (e_{uv,\phi _i}^{l})}{\sum _{k \in \mathcal {N}_{u,\phi _i}} \exp (e_{uk,\phi _i}^{l})} \end{aligned}$$

where $\sigma$ denotes the LeakyReLU activation function, *l* denotes the layer index of the multi-layer HAN, $\textbf{h}_u^{l-1}$ denotes the embedding of node *u* at the previous layer, $\textbf{a}_{\phi _i}^l$ denotes the trainable node-level attention vector. Based on the attention scores between node *u* and its meta-path based neighbors, the embeddings can be fused as:

9 $$\begin{aligned} \textbf{z}_{u,\phi _i}^l = \sigma \left( \sum _{v \in \mathcal {N}_{u,\phi _i}} \alpha _{uv,\phi _i}^l \cdot \textbf{h}_v^{l-1} \right) \end{aligned}$$

To further enhance the expressive capacity of node-level aggregation, a multi-head attention mechanism can also be adopted, which can be formulated as:

10 $$\begin{aligned} \textbf{z}_{u,\phi _i}^l = [\textbf{z}_{u,\phi _i,1}^l \parallel \cdots \parallel \textbf{z}_{u,\phi _i,h}^l] \end{aligned}$$

where *h* denotes the number of attention heads.

After the node-level attention mechanism, each node obtains a set of representations, with each representation corresponding to a specific meta-path. Subsequently, the semantic-level attention mechanism is used to aggregate the representations corresponding to different meta-paths. Given the meta-path set $\{\phi _1,\phi _2,\cdots ,\phi _P\}$, the attention score $\beta _{\phi _i}$ for each meta-path $\phi _i$ can be calculated as:

11 $$\begin{aligned} \omega _{\phi _i}^l=\frac{1}{|\mathcal {V}|} \sum _{u \in \mathcal {V}} [\textbf{q}^l]^{\top } \cdot \tanh (\textbf{W}^l \cdot \textbf{z}_{u,\phi _i}^l + \textbf{b}^l) \end{aligned}$$

12 $$\begin{aligned} \beta _{\phi _i}^l = \frac{\exp (\omega _{\phi _i}^l)}{\sum _{j=1}^{P} \exp (\omega _{\phi _j}^l)} \end{aligned}$$

where $\textbf{q}^l$, $\textbf{W}^l$, $\textbf{b}^l$ denote the trainable semantic-level attention vector, the trainable weight matrix, and the trainable bias term, respectively. Finally, the embeddings of each meta-path are integrated as:

13 $$\begin{aligned} \textbf{h}_u^{l} = \sum _{i=1}^{P} \beta _{\phi _i}^l \cdot \textbf{z}_{u,\phi _i}^l \end{aligned}$$

After completing the aggregation across all *L* layers, the embeddings of each drug pair $\{d_1,d_2\}$ from the final layer are concatenated and fed into an MLP to obtain the latent DDI embedding for subsequent prediction tasks, which can be formulated as:

14 $$\begin{aligned} \textbf{m}_{d_1 d_2}^{\text {ddi}} = \text {MLP}([\textbf{h}_{d_1}^{L} \parallel \textbf{h}_{d_2}^{L}], n_2) \end{aligned}$$

where the MLP is composed of several fully connected layers, the ReLU activation function, and batch normalization, and $n_2$ denotes the number of network layers. An MLP composed of *n* network layers can be formulated as:

15 $$\begin{aligned} \begin{aligned} \text {MLP}(\textbf{x},n)&=\text {FC}_n \bigl ( \psi _{n-1}(\cdots \psi _1(\textbf{x})) \bigr ) \\ \psi _i(\textbf{x})&=\text {ReLU}\bigl (\text {BN}_i(\text {FC}_i(\textbf{x}))\bigr ) \end{aligned} \end{aligned}$$

#### DDI decoder

As shown in Fig.  2D, DDI Decoder receives DDI latent embeddings generated by DDI Encoder and predicts the DDI categories of drug pairs through an MLP. Given a drug pair $\{d_1,d_2\}$, the DDI category prediction process can be expressed as:

16 $$\begin{aligned} \hat{y}_{d_1 d_2}^{\text {ddi}} = \text {MLP}(\textbf{m}_{d_1 d_2}^{\text {ddi}}, n_3) \end{aligned}$$

where $n_3$ denotes the number of network layers.

Note that the number of samples in each class is imbalanced, making it difficult to train DDI Decoder on such a multi-class classification task. To mitigate this issue, the focal loss [38] is employed to improve the model’s ability to focus on hard-to-classify categories. The focal loss of a sample for its true class can be represented as:

17 $$\begin{aligned} \text {FL}(p_t)=-\alpha _t(1-p_t)^\gamma \log (p_t) \end{aligned}$$

where $p_t$ denotes the predicted probability for its true class *t*, $\alpha _t$ denotes the balancing factor that adjusts the importance of true class *t*, $\gamma$ denotes the focusing parameter which controls how much focus is placed on hard-to-classify examples. In the experiments, $\alpha _t$ is set to 1 for all classes and $\gamma$ is set to 2.

#### Predictor

Predictor shown in Fig.  2E is responsible for predicting synergy scores by using the features of biological entities and DDI latent embeddings. To process the features of biological entities, Predictor is equipped with two MLPs that receive features, where one is responsible for receiving the cell line features, and the other is responsible for receiving the drug features, thereby extracting hidden biological patterns for cell lines and drugs. For each cell line *c* and drug *d*, the feature processing can be formulated as:

18 $$\begin{aligned} \begin{aligned} \textbf{m}_{c}^{\text {cell}}&= \text {MLP}(\textbf{x}_{c}^{\text {cell}},n_4) \\ \textbf{m}_{d}^{\text {drug}}&= \text {MLP}(\textbf{x}_{d}^{\text {drug}},n_5) \end{aligned} \end{aligned}$$

where $n_4$ and $n_5$ denote the number of layers of the MLP for cell line feature processing and drug feature processing, respectively.

The processed drug features $\textbf{m}_{d}^{\text {drug}}$ are then integrated with DDI latent embeddings through the multi-head attention mechanism [39], enabling the DDI embeddings to capture not only drug–drug interactions but also the intrinsic properties of the individual drugs. Specifically, the processed drug features are used as the query, while the DDI latent embeddings serve as the key and value. In this way, the drug’s intrinsic information is utilized to weight the dimensions of the DDI latent embeddings, thereby establishing a bridge between intrinsic and associated drug information. The attention output is given by:

19 $$\begin{aligned} \text {Attention}(\textbf{Q},\textbf{K},\textbf{V})=\text {softmax}(\frac{\textbf{Q}\textbf{K}^{\top }}{\sqrt{d_k}})\textbf{V} \end{aligned}$$

where $\textbf{Q}$, $\textbf{K}$, $\textbf{V}$ are the query, key and value, respectively, and $d_k$ denotes the dimension of the key. Building upon the attention mechanism defined in Equation (19), the multi-head attention mechanism enhances the model’s ability to focus on different representation subspaces simultaneously. For each head *i*, the attention scores can be calculated as:

20 $$\begin{aligned} \text {head}_i=\text {Attention}(\textbf{Q}\textbf{W}^{Q}_{i},\textbf{K}\textbf{W}^{K}_{i},\textbf{V}\textbf{W}^{V}_{i}) \end{aligned}$$

where $\textbf{Q}\textbf{W}^{Q}_{i}$, $\textbf{K}\textbf{W}^{K}_{i}$, and $\textbf{V}\textbf{W}^{V}_{i}$ transform the original $\textbf{Q}$, $\textbf{K}$, $\textbf{V}$ into a unique subspace for the *i*-th attention head, respectively. Finally, multi-head attention output is given by:

21 $$\begin{aligned} \text {MHA}(\textbf{Q},\textbf{K},\textbf{V}) = [\text {head}_1 \parallel \text {head}_2 \parallel \dots \parallel \text {head}_h]\textbf{W}^O \end{aligned}$$

where $\textbf{W}^O$ projects the concatenated outputs from all the attention heads back to the original dimensionality, thereby producing the fused embeddings $\textbf{m}^\text {ddi}_d$ for drug *d*.

Finally, the learned features of the drugs and cell lines, the DDI latent embeddings related to the drug pair, and the weighted DDI latent embeddings are concatenated and fed into a final MLP to predict the synergy score. For a given drug pair $\{d_1,d_2\}$ and a cell line *c*, the synergy score prediction process can be expressed as:

22 $$\begin{aligned} \hat{y}^{\text {syn}} = \text {MLP}([\textbf{m}_{c}^{\text {cell}} \parallel \textbf{m}_{d_1}^{\text {drug}} \parallel \textbf{m}_{d_2}^{\text {drug}} \parallel \textbf{m}_{d_1}^{\text {ddi}} \parallel \textbf{m}_{d_2}^{\text {ddi}} \parallel \textbf{m}_{d_1 d_2}^{\text {ddi}}], n_6) \end{aligned}$$

where $n_6$ denotes the number of layers of the final MLP in Predictor.

Mean squared error (MSE) is adopted as the loss to quantify the gap between the true and predicted values, which can be expressed as:

23 $$\begin{aligned} \text {MSE} = \frac{1}{N}\sum _{i=1}^{N}(y_i^{\text {syn}}-\hat{y}_i^{\text {syn}})^2 \end{aligned}$$

where *N* denotes the number of samples, and $y_i^{\text {syn}}$ and $\hat{y}_i^{\text {syn}}$ denote the true value and predicted value of the synergy score, respectively.

## Results

### Experimental setup

HGTSynergy is compared with other synergy prediction methods on the synergy score prediction task, with experiments conducted on the O’Neil dataset. The baseline methods include seven DL methods, DeepSynergy [12], DeepDDS [15], HypergraphSynergy [16], PRODeepSyn [17], DTSyn [21], MCDSP [26], and DGSS [40]. For models originally designed for classification tasks, such as DeepDDS and HypergraphSynergy, minor modifications are made to their modules and loss functions before reproducing their experiments. Since the core modules of HGTSynergy are built upon GNN-based architectures, GNN-based methods are primarily selected for comparison. For other types of approaches, the experiments include DeepSynergy, the most representative FNN-based method, and DTSyn, a Transformer-based method that is similar to HGTSynergy in its focus on capturing interactions between different drugs.

In the comparison experiments, a 5-fold nested cross-validation is performed on the O’Neil dataset following the approach outlined by Preuer et al [12]. The experiments divide the O’Neil dataset into five folds according to the Leave Drug Combinations Out strategy, ensuring that each drug combination appears only in one fold of the data, and then perform nested cross-validation. The details of dataset division are described in Additional file 4: S4. It should be noted that to eliminate the order dependency between each pair of drugs, for each quadruple (Drug A, Drug B, Cell Line, Synergy Score) in the synergy score dataset, a corresponding sample (Drug B, Drug A, Cell Line, Synergy Score) is generated for training.

For the drug autoencoder, HGTSynergy employs a fully connected encoder with layer dimensions of (1387, 512, 128), and a symmetric architecture is used for the decoder. After pre-training, the parameters of the autoencoder are fixed and the encoder is used in subsequent training stages to reduce the dimensionality of drug features. When training HGTSynergy, the number of neurons in the hidden fully connected layer of Predictor’s final MLP is selected from {2048, 4096, 8192}, and the remaining hyperparameters are fixed. A mini-batch approach is used, setting the batch size to 512 and the number of training epochs to 40. All the hyperparameters used in the experiments are listed in Additional file 4: S1.

Models are evaluated on both the synergy regression task and the synergy classification task. In this paper, the evaluation metrics used for the synergy score regression task include mean squared error (MSE), root mean squared error (RMSE), and Pearson correlation coefficient (PCC), which are employed to measure the deviation between the true values and the predicted values. Additionally, the evaluation metrics for the synergy classification task include the area under the receiver operating characteristic curve (ROC-AUC), the area under the precision–recall curve (PR-AUC), accuracy (ACC), precision (PREC), and Cohen’s Kappa.

### Method comparison

**Fig. 4:**
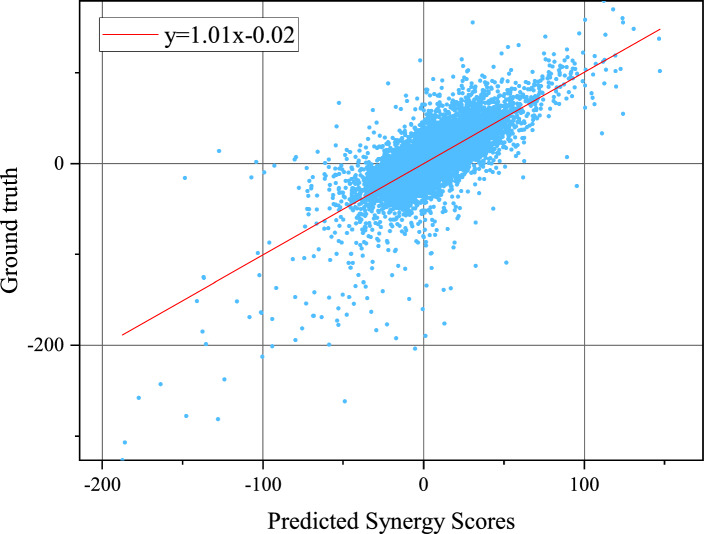
Scatter plot of the predicted synergy scores and the ground truth. The straight line is fitted using the least squares regression, whose slope is 1.01 and the intercept is $-$0.02 (p value $< 1e-5$)

Table 1 presents the performance comparison of HGTSynergy with seven DL models on the regression task, including PRODeepSyn, HypergraphSynergy, DeepSynergy, DeepDDS, DTSyn, MCDSP, and DGSS. The results indicate that HGTSynergy consistently achieves superior performance in predicting both Loewe and Bliss synergy scores, demonstrating the lowest MSE and RMSE, along with the highest PCC compared to baseline methods. For Loewe synergy score prediction, compared to PRODeepSyn, DeepSynergy, DGSS, HypergraphSynergy, MCDSP, DeepDDS, and DTSyn, HGTSynergy reduces RMSE by 0.18, 1.00, 1.67, 1.88, 2.68, 2.81, and 7.50, respectively. It also achieves the highest PCC value of 0.75, with improvements of 0.02, 0.10, 0.06, 0.08, 0.09, 0.17 over DeepSynergy, DGSS, HypergraphSynergy, MCDSP, DeepDDS, and DTSyn, respectively. For Bliss synergy score prediction, HGTSynergy reduces RMSE by 0.07, 0.57, 2.54, 2.51, 3.53, 1.56, and 2.62 compared to PRODeepSyn, DeepSynergy, DGSS, HypergraphSynergy, MCDSP, DeepDDS, and DTSyn, respectively. The PCC values are improved by 0.01, 0.08, 0.03, 0.50, 0.14, 0.21, and 0.26, respectively. Moreover, Fig.  4 illustrates the correlation between the true values and the predicted values based on Loewe synergy scores, fitted by least squares regression. The slope of the fitted line is 1.01, and the intercept is $-$0.02, indicating a strong linear correlation between HGTSynergy’s predicted values and the true values.

**Table 1:** Results of the method comparison on the regression task for predicting Loewe and Bliss synergy scores

Method	MSE	95% confidence interval	RMSE	PCC
Loewe				
HGTSynergy	**222.83**$\boldsymbol{\pm }$**22.01**	[195.50, 250.16]	**14.91**$\boldsymbol{\pm }$**0.77**	**0.75**$\boldsymbol{\pm }$**0.02**
PRODeepSyn	229.49±42.81	[176.34, 282.64]	15.09±1.37	**0.75**$\boldsymbol{\pm }$**0.02**
DeepSynergy	255.49	–	15.91±1.56	0.73±0.04
DGSS	276.11±37.17	[229.96, 322.27]	16.58±1.15	0.65±0.03
HypergraphSynergy	282.36±21.91	[255.16, 309.56]	16.79±0.67	0.69±0.03
MCDSP	311.59±50.47	[248.92, 374.26]	17.59±1.43	0.67±0.02
DeepDDS	315.07±35.76	[270.67, 359.47]	17.72±1.04	0.66±0.04
DTSyn	506.34±93.47	[390.28, 622.40]	22.41±2.04	0.58±0.03
Bliss				
HGTSynergy	**25.18**$\boldsymbol{\pm }$**2.35**	[22.27, 28.10]	**5.01**$\boldsymbol{\pm }$**0.24**	**0.75**$\boldsymbol{\pm }$**0.02**
PRODeepSyn	25.89±2.86	[22.34, 29.44]	5.08±0.29	0.74±0.02
DeepSynergy	31.25±3.44	[26.97, 35.52]	5.58±0.31	0.67±0.02
DGSS	57.20±6.51	[49.11, 65.29]	7.55±0.43	0.72±0.03
HypergraphSynergy	56.69±4.03	[51.68, 61.69]	7.52±0.27	0.25±0.02
MCDSP	73.22±9.90	[60.93, 85.51]	8.54±0.58	0.61±0.04
DeepDDS	43.30±3.79	[38.60, 48.01]	6.57±0.30	0.54±0.02
DTSyn	58.41±5.53	[51.54, 65.28]	7.63±0.36	0.49±0.03

Values of MSE, RMSE, and PCC are mean ± 1 standard deviation. The best results are shown in bold

Since many previous studies have simplified the synergy prediction problem into a binary classification problem, the true and predicted Loewe synergy scores are also binarized using a threshold of 30 by following Preuer et al. [12] to perform the classification task, and the results are presented in Table 2. Additionally, experiments with different thresholds are also conducted, and the results are presented in Additional file 4: S6. In Table 2, HGTSynergy achieves the highest values in ROC-AUC, PR-AUC, ACC and Kappa, and HypergraphSynergy achieves the highest PREC. The results indicate that HGTSynergy also demonstrates leading performance in the synergy classification task over other methods. To conclude, the improved performance of HGTSynergy can be attributed to learning latent knowledge from a large DDI dataset using a tailored pre-training task. Note that in the comparative methods, some approaches such as DTSyn and HypergraphSynergy also model relationships among drugs, but they do not train on an additional DDI dataset using a pre-defined task, and instead attempt to capture DDI relationships only from the synergy dataset, which results in limited improvement in synergy prediction performance. By contrast, HGTSynergy incorporates a substantial DDI supervised dataset to model relationships between drugs and employs a novel transfer learning framework with appropriate model construction to comprehensively capture DDI information to fully utilize this large volume of supervised data, thereby enhancing drug synergy prediction performance.

**Table 2:** Results of the method comparison on the classification task

Method	ROC-AUC	PR-AUC	ACC	PREC	Kappa
HGTSynergy	**0.90**$\boldsymbol{\pm }$**0.03**	**0.63**$\boldsymbol{\pm }$**0.04**	**0.94**$\boldsymbol{\pm }$**0.01**	0.72±0.06	**0.52**$\boldsymbol{\pm }$**0.01**
PRODeepSyn	**0.90**$\boldsymbol{\pm }$**0.03**	0.63±0.05	0.93±0.01	0.72±0.06	0.51±0.03
DeepSynergy	**0.90**$\boldsymbol{\pm }$**0.03**	0.59±0.06	0.92±0.03	0.56±0.11	0.51±0.04
DGSS	0.88±0.03	0.52±0.05	0.93±0.01	0.58±0.07	0.46±0.03
HypergraphSynergy	0.88±0.04	0.57±0.06	0.93±0.02	**0.76**$\boldsymbol{\pm }$**0.05**	0.42±0.05
MCDSP	0.87±0.03	0.55±0.09	0.92±0.02	0.58±0.15	0.46±0.05
DeepDDS	0.89±0.03	0.59±0.09	0.93±0.02	0.73±0.11	0.46±0.03
DTSyn	0.83±0.03	0.50±0.05	0.89±0.02	0.61±0.04	0.37±0.06

Values of ROC-AUC, PR-AUC, ACC, PREC, and Kappa are mean ± 1 standard deviation. The best results are shown in bold

### Ablation study

To validate the effectiveness of HGTSynergy, several variants of the model are constructed, including HGTSynergy-NoPretrain, HGTSynergy-GCN, HGTSynergy-MLP, and HGTSynergy-Concat.

- HGTSynergy-NoPretrain: This variant retains the original model structure of HGTSynergy but excludes the pre-training stage. Instead, it performs end-to-end training directly on the synergy prediction model, whose parameters are initialized randomly.
- HGTSynergy-GCN: In this variant, the HAN model is replaced with a traditional GCN model.
- HGTSynergy-MLP: In this variant, the HAN model is replaced with an MLP.
- HGTSynergy-Concat: This variant does not utilize a model to generate DDI latent embeddings. Instead, it replaces the DDI latent embeddings with embeddings constructed by directly concatenating the features of each pair of drugs.

In ablation studies, a 5-fold nested cross-validation is also conducted using the Leave Drug Combinations Out strategy, and the results are shown in Table 3. HGTSynergy-Concat is worse than HGTSynergy and its any other variants, which indicates that using a neural network to explicitly model drug relationships is helpful to the synergy prediction task. Furthermore, HGTSynergy reduces MSE by 8.23 compared to HGTSynergy-NoPretrain, which indicates that the transfer learning strategy enables HGTSynergy to learn useful implicit knowledge from the DDI data on the pre-training task, effectively enhancing the performance of the downstream synergy prediction task. Moreover, comparing HGTSynergy with HGTSynergy-GCN and HGTSynergy-MLP, it can be observed that the performance of HGTSynergy-GCN and HGTSynergy-MLP are both worse than HGTSynergy, which demonstrates that HAN has superior fitting capabilities for DDIs compared to GCN and MLP, since the tailored structure of HAN enables the model to represent different types of DDIs, allowing the model to better understand the hidden patterns within DDIs.

**Table 3:** Results in the ablation study

Method	MSE	95% confidence interval	RMSE	PCC
HGTSynergy	**222.83**$\boldsymbol{\pm }$**22.01**	[195.50, 250.16]	**14.91**$\boldsymbol{\pm }$**0.77**	**0.75**$\boldsymbol{\pm }$**0.02**
HGTSynergy-NoPretrain	231.06±25.50	[199.40, 262.72]	15.18±0.87	0.74±0.02
HGTSynergy-GCN	229.17±26.67	[196.05, 262.29]	15.11±0.92	0.74±0.03
HGTSynergy-MLP	229.12±26.01	[196.82, 261.42]	15.11±0.90	**0.75**$\boldsymbol{\pm }$**0.02**
HGTSynergy-Concat	233.61±27.71	[199.20, 268.02]	15.25±0.95	0.74±0.03

Values of MSE, RMSE, and PCC are mean ± 1 standard deviation. The best results are shown in bold

### Impact of DDI latent embeddings on prediction performance across cell lines, drugs, and tissues

**Fig. 5:**
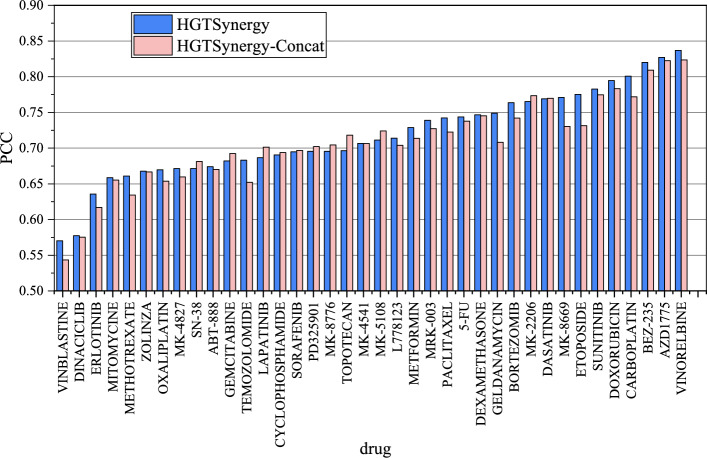
PCC values of each drug

**Fig. 6:**
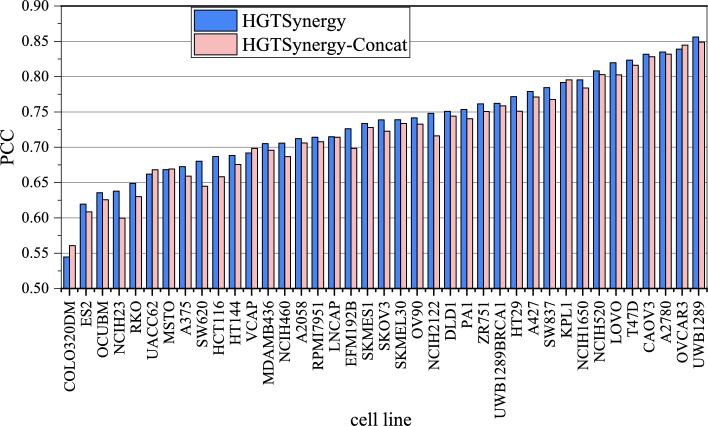
PCC values of each cell line

**Fig. 7:**
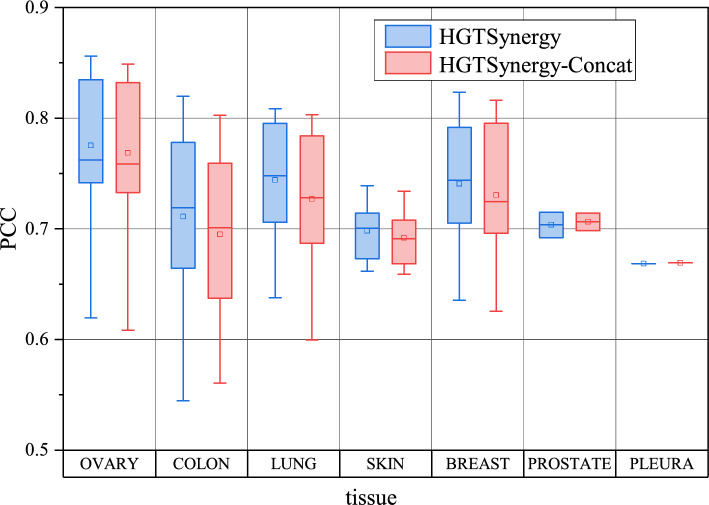
Box plots of PCC values of each tissue

To further investigate how DDI latent embeddings improve prediction performance across various drugs, cell lines, and tissues, the PCC values between true and predicted values are visualized for each drug, cell line, and tissue by comparing the results of HGTSynergy and HGTSynergy-Concat. Figure  5 demonstrates that among 38 drugs, HGTSynergy shows performance improvements on most drugs, with an increase in PCC on 27 drugs. Figure  6 also shows that among 39 cell lines, HGTSynergy shows PCC improvements on most cell lines, with an increase on 33 cell lines. In Fig.  7, boxplots are further generated to illustrate the distribution of PCC values categorized by the tissue types of cell lines, demonstrating that the DDI latent embeddings can improve PCC on most tissues. In conclusion, the performance improvement brought by learned DDI latent embeddings does not depend on individual cell lines, tissues, or drugs, and has strong potential to enhance the generalization performance of the drug synergy prediction task in general scenarios.

### Sensitivity analysis

To investigate the impact of different hyperparameter values on model performance, a sensitivity analysis is conducted on the hidden layer dimension of HAN and the learning rate. Specifically, the hidden layer dimension of HAN is selected from {20, 30, 40, 50, 60}, and the learning rate is chosen from {5e-3, 1e-3, 5e-4, 1e-4, 5e-5}. In each experiment, only one hyperparameter is varied while keeping all other hyperparameters fixed.

**Fig. 8:**
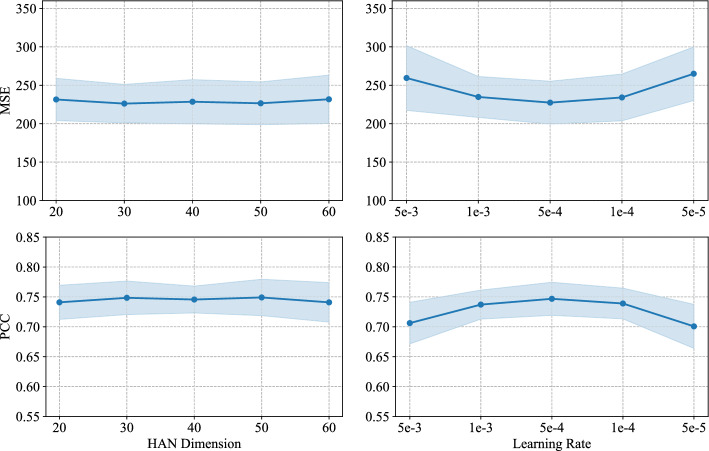
Results of sensitivity analysis. The regions in light blue indicate the range of the mean values ± 1 standard deviation

Figure  8 shows the variations in MSE and PCC with respect to the hidden layer dimension of HAN and the learning rate. As shown in the first column of Fig.  8, the prediction performance is almost unaffected by the change in the hidden layer dimension of HAN. In contrast, the second column of Fig.  8 reveals that the model is more sensitive to changes in the learning rate. When the learning rate varies within the range of 1e-3 to 1e-4, the model maintains stable performance, achieving the lowest MSE and highest PCC at 5e-4. However, outside of this range, the performance becomes highly sensitive. Therefore, it is recommended to select the learning rate around 5e-4 to ensure both stability and optimal performance.

### Visualization of DDI latent embeddings by dimension reduction

**Fig. 9:**
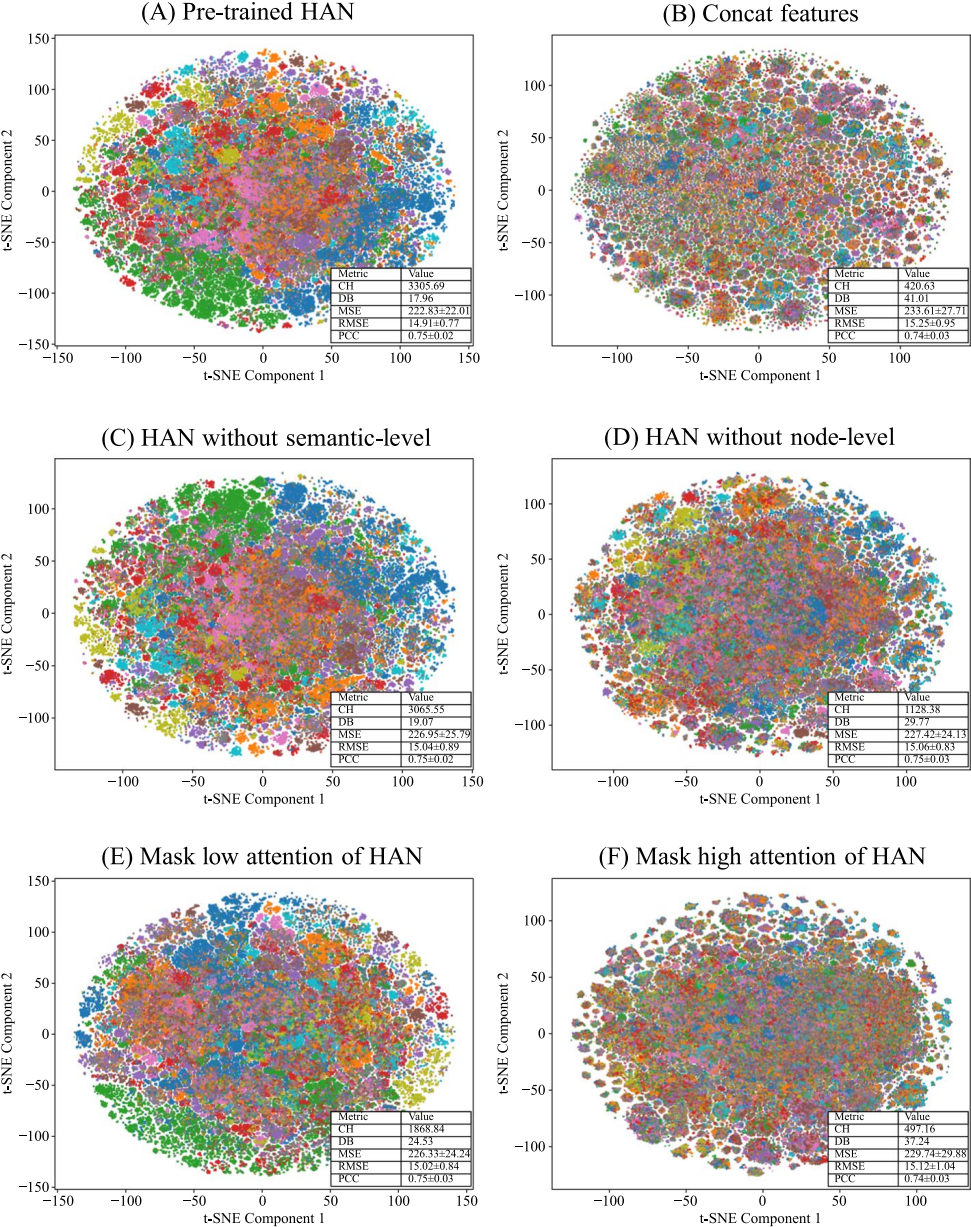
t-SNE visualization of drug-pair representations using different methods. **A** Visualization of DDI latent embeddings generated by the pre-trained DDI Encoder. **B** Visualization of concatenated features generated by the autoencoder of each drug pair. **C** Visualization of DDI latent embeddings with equal semantic-level attention scores. **D** Visualization of DDI latent embeddings with equal node-level attention scores. **E** Visualization of DDI latent embeddings where low attention scores from HAN are masked. **F** Visualization of DDI latent embeddings where high attention scores from HAN are masked

To gain deeper insight into the implicit knowledge transferred to downstream synergy prediction through transfer learning, t-SNE is used to reduce the dimensionality of the embeddings of drug pairs to 2D for visualization, as shown in Fig.  9. In each subplot of Fig.  9, each point represents the embedding of a drug pair, and different colors denote different DDI categories. This experiment evaluates the quality of DDI embeddings by observing their clustering patterns. If the embeddings form well-separated clusters, it implies that the drug pair representations can effectively discriminate between different types of DDIs, indicating high-quality DDI embeddings. In addition, this experiment employs the Calinski–Harabasz index (CH) and the Davies–Bouldin index (DB) to evaluate the clustering performance. A good clustering pattern is expected to exhibit a high CH and a low DB. Meanwhile, this experiment also compares the regression performance of different model variants on the downstream synergy score prediction task, including MSE, RMSE and PCC.

Figure  9A presents the visualization of DDI latent embeddings generated by the pre-trained HAN, which achieves the best CH and DB scores as well as the best regression performance on the downstream prediction task. In Fig.  9B, drug features generated by the autoencoder are concatenated in pairs to form DDI representations. The results show that different types of DDIs are mixed and disorganized, yielding the worst CH and DB scores as well as the worst regression performance on the downstream task, which demonstrates that the DDI latent embeddings extracted by HAN are of higher quality than the concatenated raw features of individual drugs.

To further investigate the contribution of the two-level attention mechanisms in HAN to the generation of high-quality DDI latent embeddings, the semantic-level aggregation mechanism and the node-level aggregation mechanism are individually removed, and the resulting embeddings are subsequently visualized. In Fig.  9C, the semantic-level attention scores within HAN are set to equal values, meaning the model cannot differentiate the importance of different DDI categories. Although the clustering visualization in Fig. 9C appears similar to that in Fig.  9A, both the CH and DB scores are worse, and the variant exhibits inferior regression performance on the downstream task compared to the original version, indicating that semantic-level aggregation in HAN helps the model generate higher-quality DDI latent embeddings. In Fig. 9D, the node-level attention scores in HAN are set to equal values, preventing the model from distinguishing the importance of meta-path based neighbors. The clustering result in this case is visibly worse, accompanied by significantly lower CH and higher DB scores, and it also achieves worse regression performance on the downstream task compared to that shown in Fig.  9A, suggesting that node-level aggregation under different meta-paths also contributes to the generation of high-quality DDI latent embeddings.

To further analyze the rationality of the attention score distribution in HAN, an attention-based masking experiment is conducted to examine how different attention regions affect the quality of the generated DDI latent embeddings. Specifically, features corresponding to different attention score ranges are masked based on their ranking. In Fig. 9E, the lowest 50% attention scores are masked, while in Fig.  9F, the highest 50% attention scores are masked, resulting in degraded graph node embeddings. As shown in the results, Fig.  9E yields a CH score of 1868.84, a DB score of 24.53, and an MSE of 226.33, whereas Fig.  9F yields a CH score of 497.16, a DB score of 37.24, and an MSE of 229.74. It is evident that the clustering performance in Fig.  9E is better than that in Fig.  9F, meanwhile achieving better performance of the downstream task. This indicates that high attention scores contribute more substantially to the generation of high-quality DDI latent embeddings, which demonstrates that the pre-trained HAN learns a meaningful and effective distribution of attention weights, allocating greater focus to more informative parts.

In summary, the visualization results demonstrate that using the pre-trained HAN facilitates the learning of clustering patterns in DDIs. Both node-level aggregation and semantic-level aggregation contribute to this process. Moreover, the attention scores learned by HAN exhibit a high degree of rationality, enabling the model to focus more effectively on the most informative parts. As a result, the hidden patterns revealed by this clustering can be transferred to the downstream synergy score prediction model during the fine-tuning stage of transfer learning, providing valuable information for the synergy prediction task.

### Case study

By applying HGTSynergy to predict the synergy scores of samples outside the O’Neil dataset, it can be found that many of the predicted results are consistent with previous studies. Guichard et al. [41] observed that the combination of 5-FU and SN-38 exhibited an additive effect on the HT-29 cell line, which agrees with the predicted synergy score of $-$1.04. Kano et al. [42] found that the combination of Paclitaxel and SN-38 exhibited an additive effect on the PA1 cell line. The predicted synergy score of 1.59 is consistent with this finding. Additionally, to further demonstrate the superiority of HGTSynergy, its prediction results are compared with those of PRODeepSyn in several cases. Joppert et al. [43] conducted in vitro experiments and found that the combination of Topotecan and Gemcitabine exhibited an additive effect in lung cancer cells. On seven lung cell lines including A427, NCIH1650, NCIH2122, NCIH23, NCIH460, NCIH520, and SKMES1, the predicted synergy scores by HGTSynergy are 3.22, 1.22, 2.80, $-$4.34, 2.02, 8.91, and $-$8.63, respectively, while the corresponding predictions by PRODeepSyn are 9.74, $-$2.15, 4.06, $-$8.42, $-$1.62, 9.22, and $-$14.77. It can be observed that both methods do not show strong synergistic or antagonistic effects, which aligns with the additive effect reported in the literature, but the predictions of HGTSynergy are closer to zero compared to those of PRODeepSyn, indicating that HGTSynergy captures the additive effect more accurately. Moreover, Bahadori et al. [44] conducted in vitro experiments on the combination of Topotecan and Vinblastine in colon tissue and reported a synergistic effect. The predicted synergy scores of HGTSynergy for this drug combination on eight colon cell lines including COLO320DM, DLD1, HCT116, HT29, LOVO, RKO, SW620, and SW837 are 7.22, 11.91, 6.89, 2.86, 12.94, 17.93, 34.38, and 18.06, respectively, while those of PRODeepSyn are 6.80, 5.49, 0.03, $-$6.04, 5.77, $-$19.32, 26.87, and 3.72. It can be seen that HGTSynergy consistently predicts a synergistic effect for the Topotecan and Vinblastine combination across all colon cell lines, whereas PRODeepSyn shows less consistent synergistic predictions and even predicts antagonistic effects in some cases. Therefore, these cases suggest that HGTSynergy outperforms PRODeepSyn in certain scenarios, which can be attributed to HGTSynergy’s ability to better capture the underlying mechanisms of drug interactions through heterogeneous graph modeling and transfer learning.

## Discussion and conclusion

This paper proposes HGTSynergy for predicting synergistic anti-cancer drug combinations. Within a transfer learning framework, it first learns prior knowledge from a large-scale DDI heterogeneous graph through a tailored task, and then fine-tunes the model using a small-scale synergy score dataset. The performance advantages of HGTSynergy are reflected in its construction of a DDI heterogeneous graph that fully accounts for multiple DDI categories and its use of HAN to effectively capture the structural features of the drug network. Meanwhile, transfer learning enables the network to benefit from DDIs by capturing complex drug associations through a tailored pre-training task and providing them to the downstream task to improve drug synergy prediction performance.

The experiments conducted on the O’Neil dataset demonstrate that HGTSynergy outperforms other methods in drug synergy prediction on both the synergy regression task and the synergy classification task. In addition, the ablation studies provide compelling evidence that using HAN to capture the hidden patterns of diverse DDIs is superior to other traditional networks. It also demonstrates that the proposed transfer learning strategy can significantly enhance synergy prediction performance. Furthermore, it can be found that the DDI latent embeddings generated in the proposed framework improve synergy prediction performance on the vast majority of cell lines, tissues, and drugs, suggesting that the proposed method has the potential to achieve good generalization performance in more general scenarios. The visualization results demonstrate the good interpretability of DDI latent embeddings. This work also conducts experiments to analyze the hyperparameter sensitivity of HGTSynergy, and the results indicate that the model performance is insensitive to the hidden layer dimension of the HAN, but sensitive to the learning rate, therefore offering valuable guidance for hyperparameter setting during model optimization. Additionally, the case study demonstrates that the predictions of HGTSynergy align with the outcomes of previous studies in many instances, which demonstrates the practical value of HGTSynergy. Overall, HGTSynergy represents a good advancement in drug synergy prediction.

HGTSynergy still has limitations. HGTSynergy does not model the microscopic molecular structures of drugs. Instead, the molecular fingerprints and descriptors are concatenated into vectors and directly used as input features. This design limits the model’s interpretability at the molecular level. Future work will investigate drug molecular structure modeling strategies, inspired by prior work that has already introduced molecular-level representations for drugs [40, 45, 46]. Additionally, when constructing the DDI heterogeneous graph, this work only considers the DDIs included in DrugBank, while many DDIs are not yet recorded or determined, which may cause the DDI heterogeneous graph to not fully reflect all interaction relationships in the drug network. Recently, many studies [47, 48] have explored how to predict DDI relationships, and incorporating such tasks to obtain a more complete DDI heterogeneous graph may help to further improve synergy prediction performance in future work.

In summary, the findings suggest the use of a transfer learning method based on a DDI heterogeneous graph to search for synergistic drug combinations and provide a possible reference for studying the synergistic mechanisms of anticancer drug combinations. HGTSynergy has the potential to become a powerful tool for pre-screening synergistic anti-cancer drug combinations.

## Additional file

Additional file 1

Additional file 2

Additional file 3

Additional file 4

## Data Availability

The source code and data are available at https://github.com/Bakers-Lab/HGTSynergy. Drug SMILES representations and drug-drug interaction data are obtained from the DrugBank database (version 5.1.12; https://go.drugbank.com/), gene expression data are retrieved from the ArrayExpress database (accession number: E-MTAB-3610; https://www.ebi.ac.uk/biostudies/arrayexpress), gene mutation data are collected from the COSMIC database (https://cancer.sanger.ac.uk/cosmic/), protein-protein interaction data are sourced from the STRING database (version 10.5; https://cn.string-db.org/), Loewe synergy scores are obtained from https://github.com/KristinaPreuer/DeepSynergy, and Bliss synergy scores are obtained from https://github.com/zhangpengxx/MGAE-DC.

## Abbreviations

HTS: High-throughput screening
ML: Machine learning
DL: Deep learning
FNN: Feedforward neural network
GNN: Graph neural network
DDI: Drug-drug interaction
SOTA: State-of-the-art
PPI: Protein-protein interaction
GCN: Graph convolutional network
HAN: Heterogeneous graph attention network
MLP: Multi-layer perceptron
MSE: Mean squared error
RMSE: Root mean squared error
PCC: Pearson correlation coefficient
ROC-AUC: Area under the receiver operating characteristic curve
PR-AUC: Area under the precision–recall curve
ACC: Accuracy
PREC: Precision
CH: Calinski–Harabasz index
DB: Davies–Bouldin index
