# Supplementary material for: Hgtsynergy: a transfer learning method for predicting anticancer synergistic drug combinations based on a drug-drug interaction heterogeneous graph
Source: BMC Bioinformatics. 2026 Jan 6;27:35. doi: 10.1186/s12859-025-06360-5 (PMC12870253; doi:10.1186/s12859-025-06360-5)
Supplement: Supplementary file 4 — Additional file 4 [file 12859_2025_6360_MOESM4_ESM.pdf]

HGTSynergy: a transfer learning method for predicting anticancer synergistic drug combinations based on a drug-drug interaction heterogeneous graph

Xiaowen Wang, Yanming Huang, Hongming Zhu,
Dongsheng Mao, Xiaoli Zhu, Qin Liu

Supplementary Explanations and Data

S1. Details of Hyperparameters

Due to the high dimensionality of raw drug features, an autoencoder is employed for dimensionality reduction. The detailed hyperparameter settings of the autoencoder are listed in the table below:

Table 1: Hyperparameter Settings for Drug AutoEncoder

Hyperparameter	Value
layer_dim	(1387, 512, 128, 512, 1387)
learning_rate	5e-4
batch_size	64
epochs	1000
act_func	ReLU
loss_func	MSELoss
optimizer	Adam
ReduceLROnPlateau_factor	0.1
ReduceLROnPlateau_patience	5
ReduceLROnPlateau_threshold	0.0001

The detailed hyperparameters of the Cell StateEncode are as follows:

Table 2: Hyperparameter Settings for Cell StateEncoder

Hyperparameter	Value
gnn_layers	2
gnn_hidden_dim	128
learning_rate	1e-3
gene_dim	384
mutation_dim	384
optimizer	Adam
act_func	ReLU
early_stop_patience	50
epochs	1000

The detailed hyperparameter settings for the pre-training stage are shown in the table below:

Table 3: Hyperparameter Settings for Pre-training Stage

Hyperparameter	Value
learning_rate	1e-3
batch_size	1024
epochs	1000
act_func	ReLU
loss_func	FocalLoss
gnn_layers	2
gnn_hidden_dim	50
optimizer	AdamW

After the pre-training is completed, the parameters of the pre-trained model are transferred to the downstream synergy prediction model and subsequently fine-tuned. The detailed hyperparameter settings for the fine-tuning stage are listed in the table below:

Table 4: Hyperparameter Settings for Fine-tuning Stage

Hyperparameter	Value
learning_rate	1e-4
batch_size	512
epochs	40
act_func	ReLU
loss_func	MSELoss
dropout_han	0
dropout_mlp	0.2
gnn_layers	2
gnn_hidden_dim	40
predictor_fc_hidden_dim	selected from {2048, 4096, 8192}
attention_heads	4
optimizer	AdamW
ReduceLROnPlateau_factor	0.1
ReduceLROnPlateau_patience	5
ReduceLROnPlateau_threshold	0.1
weight_decay (L2)	1e-3

S2. Details of Cell Line Feature Construction

The implementation details of the cell line feature construction method in this study are based on the approach proposed in PRODeepSyn, with all imple-

mentations built upon the open-source code repository provided by the original paper: `https://github.com/TOJSSE-iData/PRODeepSyn`.

PRODeepSyn integrates three types of data containing gene expression data, gene mutation data and interactions between gene expression products to construct embeddings for cell lines. The gene expression data is downloaded from the ArrayExpress database (accession number: E-MTAB-3610). A total of 3739 informative genes are first summarized with the Factor Analysis for Robust Microarray Summarization method and then processed by the z-score normalization method. Gene mutation data of cell lines are obtained from the COSMIC cell lines project. We remove data whose mutation type is coding silent or unknown and retain the mutation data of 12,333 genes for 39 cell lines. The gene mutation data of each cell line is represented as a 12,333-dimensional binary-valued vector. According to whether the cell line is mutated on a gene, the corresponding element of the vector is 0 or 1. Interactions between gene expression products are collected from the PPI network contained in the STRING database. We ignore the interactions whose combined scores are lower than 0.7 in STRING and retain a total of 839,522 interactions between 17,161 proteins. We associate the nodes in the PPI network with gene expression data and gene mutation data via gene identifiers and symbols.

The detailed steps for constructing cell line features are as follows:
1. Treat gene expression and gene mutation as the gene explicit state $\mathbf{o}_j^{\mathrm{Exp}}$ and $\mathbf{o}_j^{\mathrm{Mut}}$; build a PPI network (proteins as nodes, interactions as edges).
2. Initialize a trainable vector $\mathbf{c}_j$ for each cell line j.
3. Initialize the Projector (a fully connected layer) to perform a project transformation f.
4. Run a GCN on the PPI graph to obtain node embeddings; use these embeddings as the gene hidden state $\mathbf{Z}$.
5. For each cell line j, approximate its gene explicit state vector by the dot product between the projected gene hidden state $\mathbf{Z}$ and the cell line state $\mathbf{c}_j$, which can be denoted as $\mathbf{o}_j^t = f(\mathbf{Z}) \cdot \mathbf{c}_j, t \in \{\mathrm{Exp}, \mathrm{Mut}\}$.
6. Train the GCN, the Projector, and the cell line state vectors by minimizing the reconstruction error of the gene explicit state (expression and mutation); the learned cell line state $\mathbf{c}_j$ serves as the final cell line embedding for downstream prediction.

S3. Details of the O'Neil dataset

The O'Neil dataset comprises 23,052 samples, where each sample consists of two drugs and a cell line, while in our study, we used 22,737 samples after removing duplicate entries. The dataset covers 583 distinct combinations, each tested against 39 human cancer cell lines derived from 7 different tissue types. Pairwise combinations were constructed from 38 diverse anticancer drugs (14 experimental and 24 approved), of which 22 were tested exhaustively in combination (the "exhaustive" set), while the remaining 16 (the "supplemental" set) were tested only in combination with those of the exhaustive set.

The experiments used Loewe Additivity and Bliss Independence synergy scores computed from the O'Neil dataset as prediction labels. The distributions of these two synergy scores are shown in Figure 1, and key statistics of the dataset are summarized in Table 5. As shown in Figure 1, the synergy scores exhibit an uneven distribution. The synergy scores of most samples are close to 0, indicating additive effects, while only a small proportion exhibit synergistic or antagonistic effects. For classification performance evaluation, we selected a threshold of 30 to binarize the Loewe synergy scores, such that the top 10% of synergy scores were labeled as positive samples, and the remaining as negative samples.

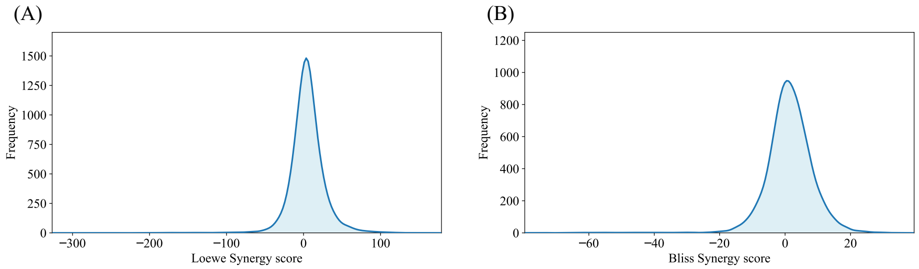

Figure 1: Data distributions of the datasets used in this paper. (A) Distribution of the Loewe synergy scores in the O'Neil dataset. (B) Distribution of the Bliss synergy scores in the O'Neil dataset.

Table 5: Summary statistics for Loewe Additivity and Bliss Independence synergy scores in the O'Neil dataset

**Metric**	**Loewe**	**Bliss**
Max	179.12	39.4525
Min	-326.46	-79.5925
Mean	4.89	1.3263
Std	22.70	7.5194

S4. Details of 5-fold Nested Cross-Validation

HGTSynergy is evaluated on the O'Neil dataset with 5-fold nested cross-validation. The detailed steps are as follows:

1. Split the synergy dataset into five folds according to the Leave Drug Combincations Out stragtegy, ensuring that the same drug combination appears only in one fold of the data.

2. Choose one fold as a test fold each time and note the other four folds as outer training folds.

3. Choose a hyperparameter setting for evaluation.

4. For each fold in outer training folds, choose one fold as the validation fold each time and note the other folds as inner training folds.

5. Train the model on the inner folds with the selected hyperparameter setting, and calculate the performance on the validation fold.

6. Repeat Step 4-5 until each outer training fold from the synergy dataset has been chosen as the validation fold. Calculate the mean MSE on the four outer training folds as the performance of the selected hyperparameters.

7. Repeat Step 3-6 to evaluate all hyperparameter settings and select the hyperparameter setting with the best performance.

8. Randomly split the outer training folds from the synergy dataset to 10 folds. Randomly choose one fold as a small validation fold and train the model with the other 9 folds.

9. Train HGTSynergy with the selected hyperparameter setting in Step 7. During training, the MSE on the small validation set is continuously monitored. If the MSE does not show significant improvement over several consecutive epochs, the learning rate is reduced to one-tenth of its current value. After training is completed, the model corresponding to the epoch with the best validation performance is saved.

10. Evaluate the trained model from Step 9 on the test fold selected in Step 2.

11. Repeat Step 2-10 to complete the 5-fold nested cross-validation.

S5. Complexity Analysis

The computational complexity and the number of parameters of the proposed model are analyzed in this section. For simplicity, it is assumed that the hidden dimensions of all layers within the same type of network are identical. A detailed analysis of the computational complexity and number of trainable parameters for each module in HGTSynergy is provided below:

1. The Computational Complexity

DDI Encoder: This module consists of HAN and MLP. Let the hidden dimension of HAN be d_h, the number of layers be L_h, the number of meta-paths be P, the number of nodes be V, and the number of edges be E. The computational complexity of the node-level aggregation in HAN can be expressed as:

$$O(L_h E d_h).$$

The computational complexity of the semantic-level aggregation in HAN can be denoted as:

$$O(L_h P V d_h^2).$$

In addition, the MLP component of the DDI Encoder consists of several fully connected layers. Let the hidden dimension of these layers be d_f and the number of layers be L_f. The computational complexity of the MLP can be denoted as:

$$O(L_f d_f^2).$$

Therefore, the total computational complexity of the DDI Encoder can be expressed as:

$$O(L_h(Ed_h + PVd_h^2) + L_f d_f^2).$$

It can be observed that the main computational bottleneck of the DDI Encoder comes from HAN. The computational complexity of HAN grows linearly with the number of nodes and edges in the graph. Meanwhile, HAN can be easily parallelized, as the node-level and semantic-level attention mechanisms can be parallelized across node pairs and meta-paths, respectively. Therefore, the computation of HAN is efficient, and its complexity is acceptable.

DDI Decoder: This module only contains MLP layers. With a hidden dimension of d_f and L_f layers, the computational complexity is:

$$O(L_f d_f^2).$$

Predictor: This module consists of MLP and an attention mechanism. The MLP has a complexity of:

$$O(L_f d_f^2).$$

Let the hidden dimension of the attention mechanism be d_a, the number of drugs be 2, and the number of DDIs used for attention be T. The computational complexity for attention mechanism is:

$$O(2d_a T + d_a^2 T) = O(d_a^2 T)$$

Therefore, the total computational complexity of the Predictor is:

$$O(L_f d_f^2 + d_a^2 T).$$

2. The number of trainable parameters

DDI Encoder: This module consists of HAN and MLP. In HAN, each meta-path is equipped with one attention vector for node-level aggregation, resulting in:

$$O(L_h P d_h)$$

For semantic-level aggregation, one attention matrix and two attention vectors are used, resulting in:

$$O(L_h d_h^2 + 2L_h d_h) = O(L_h d_h^2)$$

The number of trainable parameters of the MLP is:

$$O(L_f d_f^2).$$

Thus, the total number of trainable parameters of the DDI Encoder is:

$$O(L_h(Pd_h + d_h^2) + L_f d_f^2).$$

It can be observed that the number of trainable parameters in the HAN module of DDI Encoder is independent of the graph size. Therefore, the number of trainable parameters is acceptable.

DDI Decoder: Since this module only consists of MLP layers, the number of trainable parameters is:

$$O(L_f d_f^2).$$

Predictor: The number of trainable parameters of the MLP in this module is:

$$O(L_f d_f^2),$$

The number of trainable parameters of the attention mechanism is:

$$O(d_a^2).$$

Therefore, the total number of parameters in the Predictor is:

$$O(L_f d_f^2 + d_a^2).$$

S6. Experiments with Different Binary Classification Thresholds for Synergy Scores

In this study, the experiments follow the setting of DeepSynergy by using 30 as the threshold to determine whether a synergy score indicates a synergistic effect. To provide a more comprehensive evaluation of HGTSynergy's classification performance, we present the classification metrics of HGTSynergy and other models under different threshold settings (10, 20, 30) in this section. The results show that HGTSynergy achieves the best performance in terms of ROC-AUC, PR-AUC, ACC, and Kappa when the threshold is set to 30. Although HGTSynergy does not achieve the best performance at thresholds of 10 or 20, its results are still very close to the best. These findings indicate that the proposed HGTSynergy performs particularly well under higher thresholds, demonstrating that the model can make more precise predictions for drug combinations with

stronger synergistic effects.

Table 6: Results of the method comparison on the classification task across thresholds.

Method	ROC-AUC	PR-AUC	ACC	PREC	Kappa
Threshold = 10					
HGTSynergy	0.86±0.02	0.79±0.03	0.81±0.02	0.75±0.03	0.56±0.04
PRODeepSyn	**0.87±0.01**	0.80±0.03	**0.81±0.01**	0.73±0.03	**0.58±0.02**
DeepSynergy	0.86±0.01	0.77±0.03	0.80±0.02	0.74±0.03	0.54±0.03
DGSS	0.83±0.02	0.73±0.03	0.78±0.02	0.69±0.03	0.50±0.03
HypergraphSynergy	0.83±0.02	0.74±0.02	0.78±0.03	0.74±0.03	0.47±0.04
MCDSP	0.81±0.02	0.71±0.04	0.76±0.03	0.65±0.04	0.47±0.03
DeepDDS	0.84±0.02	0.76±0.03	0.79±0.02	0.71±0.04	0.52±0.03
DTSyn	0.82±0.02	**0.81±0.03**	0.73±0.04	**0.81±0.04**	0.45±0.06
Threshold = 20					
HGTSynergy	0.89±0.02	0.70±0.04	**0.88±0.02**	0.72±0.03	0.55±0.04
PRODeepSyn	**0.89±0.01**	**0.71±0.04**	**0.88±0.02**	0.72±0.04	**0.56±0.03**
DeepSynergy	0.88±0.02	0.67±0.05	0.87±0.02	0.70±0.03	0.51±0.04
DGSS	0.86±0.02	0.61±0.02	0.87±0.02	0.63±0.03	0.49±0.04
HypergraphSynergy	0.86±0.03	0.65±0.03	0.88±0.03	**0.78±0.03**	0.45±0.04
MCDSP	0.85±0.02	0.61±0.05	0.85±0.03	0.57±0.07	0.47±0.04
DeepDDS	0.87±0.02	0.67±0.05	0.87±0.03	0.69±0.07	0.51±0.04
DTSyn	0.82±0.02	0.62±0.05	0.81±0.03	0.66±0.04	0.42±0.07
Threshold = 30					
HGTSynergy	**0.90±0.03**	**0.63±0.04**	**0.94±0.01**	0.72±0.06	**0.52±0.01**
PRODeepSyn	**0.90±0.03**	0.63±0.05	0.93±0.01	0.72±0.06	0.51±0.03
DeepSynergy	**0.90±0.03**	0.59±0.06	0.92±0.03	0.56±0.11	0.51±0.04
DGSS	0.88±0.03	0.52±0.05	0.93±0.01	0.58±0.07	0.46±0.03
HypergraphSynergy	0.88±0.04	0.57±0.06	0.93±0.02	**0.76±0.05**	0.42±0.05
MCDSP	0.87±0.03	0.55±0.09	0.92±0.02	0.58±0.15	0.46±0.05
DeepDDS	0.89±0.03	0.59±0.09	0.93±0.02	0.73±0.11	0.46±0.03
DTSyn	0.83±0.03	0.50±0.05	0.89±0.02	0.61±0.04	0.37±0.06

Note: Values of ROC-AUC, PR-AUC, ACC, PREC, and Kappa are mean ± 1 standard deviation. The best results are shown in bold.
